# Diseases from a Book

**Published:** 1890-02

**Authors:** 


### DISEASES FROM A BOOK.

In 1846, a boy of eight years, the brother of the narrator's wife, was taken down with scarlet fever and died. One of the principal amusements of his illness had been looking over a large picture book. After his death this, with several other useful playthings, was packed away in a trunk. Twenty-six years later, in 1872, the sister-in-law of the editor took this trunk with her on a journey which she made to England, where he was then residing. The trunk was opened the second day after its arrival, and the picture book was taken out and presented to the editor's two-year-old son. During the next fortnight the little fellow was attacked by scarlet fever. It was a wonder to the doctors who were called in consultation how the disease had been contracted, as there had been no scarlet fever in the town for years. At last it occurred to the editor that the picture book might have transmitted the disease, and the medical men in attendance, on being told the facts connected with it, agreed that it had retained the poison for twenty-six years and communicated it to the child.—*Boston Post.*

Typhoid Fever.—It is generally conceded that nothing is more discreditable to the civilization of the nineteenth century than the existence of typhoid fever. Typhoid fever never infects the atmosphere, it never arises de novo. The causes of the disease, in order of their frequency, are as follows: First, infected water; second, infected milk; third, infected ice; fourth, digital infection; fifth, infected meat. Dr. Edson states that with the observations of the ordinary obvious precautions suggested by these conclusions, the disease should not exist.

